# Structure-function relationship of γ-secretase

**DOI:** 10.1186/1750-1326-7-S1-L5

**Published:** 2012-02-07

**Authors:** Taisuke Tomita

**Affiliations:** 1 Department of Neuropathology and Neuroscience, Graduate School of Pharmaceutical Sciences, The University of Tokyo, Tokyo, Japan

## Background

Genetic and biological studies provide strong evidence that the production and deposition of amyloid-β peptides (Aβ) contribute to the etiology of Alzheimer’s disease (AD). γ-Secretase is an unusual aspartic protease that cleaves the scissile bond within the transmembrane domain of APP to generate Aβ. This unusual enzyme is composed of a high molecular weight membrane protein complex containing presenilin, nicastrin, Aph-1 and Pen-2. Drugs that regulate the production of Aβ by inhibiting or modulating the γ-secretase activity could provide a disease-modifying effect on AD, although recent studies suggest that the γ-secretase plays important roles in cellular signaling including Notch pathway. Thus, understanding the molecular mechanism whereby the γ-secretase cleaves its substrate is a critical issue for the development of compounds that specifically regulate the Aβ-generating γ-secretase activity.

## Methods

To analyze the structure of PS, the catalytic subunit of γ-secretase, we have employed substituted cysteine accessibility method (SCAM), a biochemical method by which structures of various membrane proteins have been analyzed in a functional state. In addition, we identified the target molecule/domain of the γ-secretase inhibitors and modulators using chemical biology approach. Finally, we rationally developed novel reagents that regulate the γ-secretase activity.

## Results

We found that the hydrophilic “catalytic pore” structure of γ-secretase is formed by the transmembrane domains (TMD) 1, 6, 7 and 9 of PS1 within the membrane. Competition experiments by γ-secretase inhibitors suggest that the N-terminal region of TMD1 directly faces the hydrophilic environment within the lipid bilayer as a part of the catalytic site. Intriguingly, inhibitor binding affected water accessibility of residues at the membrane border of TMD1. Moreover, we successfully raised a novel inhibitory monoclonal antibody against γ-secretase activity, which targets the juxtamembrane region of TMD1 of PS1. Finally, we identified that GSM-1, a potent γ-secretase modulator, binds to the hydrophobic region of TMD1 and affects the catalytic pore structure.

## Conclusion

TMD1 of PS1 is a functionally critical domain for the γ-secretase activity.

